# Isolation and characterization of bacterial endophytes of *Curcuma longa* L.

**DOI:** 10.1007/s13205-016-0393-y

**Published:** 2016-02-13

**Authors:** Ajay Kumar, Ritu Singh, Akhilesh Yadav, D. D. Giri, P. K. Singh, Kapil D. Pandey

**Affiliations:** Centre of Advance Study in Botany, Banaras Hindu University, Varanasi, 221005 India

**Keywords:** Antimicrobial activity, *Curcuma longa*, Endophyte, 16S rRNA analysis, Substrate utilization

## Abstract

Fourteen endophytic bacterial isolates were isolated from the rhizome of *Curcuma longa* L. were characterized on the basis of morphology, biochemical characteristics and 16S rRNA gene sequence analysis. The isolates were identified to six strains namely *Bacillus cereus* (ECL1), *Bacillus thuringiensis* (ECL2), *Bacillus* sp. (ECL3), *Bacillus pumilis* (ECL4), *Pseudomonas putida* (ECL5), and *Clavibacter* *michiganensis* (ECL6). All the strains produced IAA and solubilized phosphate and only two strains produced siderophore (ECL3 and ECL5) during plant growth promoting trait analysis. All the endophytic strains utilized glucose, sucrose and yeast extract as a carbon source where as glycine, alanine, cystine and glutamine as nitrogen source. The strains were mostly sensitive to antibiotic chloramphenicol followed by erythromycin while resistant to polymixin B. The endophytic strains effectively inhibit the growth of *Escherichia coli*, *Klebsiella pneumoniae* and some of the fungal strain like *Fusarium solani* and *Alterneria alternata*. The strain ECL2 and ECL4 tolerated maximum 8 % of NaCl concentration where as strains ECL5 and ECL6 6 % in salinity tolerance.

**Electronic supplementary material:**

The online version of this article (doi:10.1007/s13205-016-0393-y) contains supplementary material, which is available to authorized users.

## Introduction

*Curcuma longa* L. commonly known as turmeric is a rhizotomous herb of family Zingiberaceae cultivated in Indian sub-continent and the Middle East countries. The mature dried rhizome is most common ingredient of Indian kitchen as spice and well known antiseptic, antipyretic since ancient times. The medicinal properties are assigned due to the presence of curcuminoid and sesquiterpenoid compounds. Curcumin, the most important curcuminoid, is used as antioxidant, antimicrobial anti-inflammatory andis even effective against cancer and HIV (Srimal 1997; Aggarwal et al. 2007; Aggarwal and Sung 2009). Plants interact with diverse communities of microorganisms for various purposes including growth promotion, yield enhancement and disease management. In turn, the microbes derive shelter and nutrients from the host plants (Reiter and Sessitsch 2006). Rhizosphere is the most prominent zone for the microbial interaction, while some of the bacterial species enter inside the plant tissue and resides as endophytes without causing any symptoms. During endophytic colonization the microbe resides in almost every internal part of plant ranging from tissues of the underground roots to stem, leaf, flower, fruit and seed (Hallmann et al. 1997a, b). The endophyte population varies depending upon the tissue, plant developmental stage and the surrounding environment such as season as reported in soybean (Kuklinsky-Sobral et al. 2004). Endophytes actively or passively trigger the physiological changes in the plant cell (Conrath et al. 2006). They are superior in growth promotion over the rhizobacteria owing to better adaptations against abiotic or biotic stresses (Pillay and Nowak 1997). Bacterial endophytes promote host plant growth through direct mechanisms by producing phytohormones IAA, gibberellins, cytokinins (Lee et al. 2004), phosphate solubilization (Wakelin 2004), N_2_ fixation (Compant et al. 2005) or indirectly by production of antibiotics (Ezra et al. 2004), siderophores (Lodewyckx et al. 2002) and lytic enzymes against the pathogens (Chernin and Chet 2002). Many endophytes constitute the common soil bacteria (*Pseudomonas*, *Burkholderia* and *Bacillus*) that produce diverse range of secondary metabolites, antibiotics and volatile organics to counters the deleterious effects of pathogens by mechanisms in line with the PGPR (Kloepper et al. 1999; Lodewyckx et al. 2002). In addition, endophytes also counteract the adverse effects of salinity for sustainable agriculture.

The underground turmeric rhizome favors growth of various microbial communities or endophyte, which modulates plant growth through the synthesis of biochemicals and secondary metabolites. The present study was undertaken to study the endophytic bacterial community of the turmeric rhizome and their response to the salinity stress, antimicrobial agents and substrate utilization pattern.

## Materials and methods

For the isolation of endophytic bacterial isolates, the rhizomes of *C. longa* L.(Turmeric) were collected from the Botanical garden of Banaras Hindu University, Varanasi, India (20°18′N and 80°36′E, elevation 80.71 m). Fresh and healthy rhizomes were washed thoroughly under running tap water and surface sterilized (70 % C_2_H_5_OH, 3 min, 0.5 % NaOCl, 3 min and 70 % C_2_H_5_OH, 30 s). Finally, they were washed thrice with sterile distilled water (Sun et al. 2008). Surface sterilization efficiency of the sterilizers was checked by inoculating surface sterilized unsliced rhizome on nutrient agar plate, prior to inoculation of endophytic bacteria. The surface sterilized rhizomes were air dried, sliced into thin sections and placed aseptically over nutrient agar plate Petri dishes and incubated at 30 °C for 2–4 days in bacteriological incubator. The bacterial colonies surrounding rhizome section were picked and streaked on the fresh nutrient agar for the selection of clone.

### Characterization of bacterial isolates

Endophytic bacterial isolates were characterized on the basis of colony morphology, biochemical characteristics and molecular phylogeny. The morphological and biochemical characteristics of the isolates were examined according to the Bergey’s manual of determinative Bacteriology (Kumar et al. 2015).

### 16S rRNA gene amplification and sequencing

Genomic DNA was isolated using Genei Pure™ bacterial DNA purification kit (GeNei™, Bangaluru, India) following the manufacture’s protocol. Universal eubacterial primers F-D1 5′-CCGAATTCGTCGACAACAGAGTTTGATCCTGGCTCAG-3′ and R-D1 5′-CCCGGGATCCAAGCTTAAGGAGGTGATCCAGCC-3′ (Kumar et al. 2015) were used to amplify 1500 bp region of 16S rRNA gene using a thermal cycler (BioRad, USA). Amplification products were resolved by agarose-gel electrophoresis (1.5 %), and visualized using a gel documentation system (Alfa Imager, Alfa Innotech Corporation, USA). The ampicons were purified using Genei Pure™ quick PCR purification kit (GeNei™, Bangaluru, India) and quantified at 260 nm using a spectrophotometer taking calf thymus DNA as control. The purified partial 16S rDNA amplicons were sequenced in an Applied Biosystems 3130 Genetic Analyzer (Applied Biosystems^®^, CA, and USA).

### Analysis of 16S rDNA sequences

The partial sequences of nucleotides were compared with available sequences from NCBI databases and sequences showing >99 % similarity were retrieved by Nucleotide Basic Local Alignment Search Tool (BLAST N) program available at the National Center for Biotechnology Information (NCBI) BLAST server (www.ncbi.nlm.nih.gov/BLAST).

### PGP traits analysis

#### Production of IAA

Bacteria were cultivated at 25 ± 2 °C for 48 h in the nutrient agar broth supplemented with 100–400 µg ml^−1^ of l-tryptophan were harvested through centrifugation (8000 rpm, 10 min). Supernatant (2 ml) was mixed with 2 drops of orthophospheric acid and 4 ml of the Salkowski reagent (50 ml, 35 % of perchloric acid, 1 ml 0.5 M FeCl_3_ solution) (Bric et al. 1991). Production of IAA was confirmed by the development of pink color.

#### Phosphate solubilization

The bacterial strains were inoculated at three to four places on the Pikovskaya medium containing tricalcium phosphate on nutrient agar plate and incubated at 28 ± 2 °C for 2–3 days (Pikovskaya 1948). Development of clear halo zone around the strains exhibited their positive phosphate solubilization activity.

### Siderophore production

The cultured bacterial strains were spotted on the Chrome azurol S agar plate (Schwyn and Neilands 1987). Development of yellow orange hallow zone around the bacterial spot has been considered as positive indication for siderophore production.

### Substrate utilization

Carbohydrate utilization was performed by modifying the Simmon’s Citrate medium with mixture of amino Acids (i.e. alanine, glycine, glutamine, cysteine, methionine and trptophan in equal amount), A_5_ and A_6_ trace element, multivitamin capsule and by replacing the sodium citrate with 0.2 % (w/v) of different carbohydrates. Growth was examined after fifth day of incubation in comparison with negative control. Nitrogen utilization pattern was tested by adding 0.1 % (w/v) different nitrogen source in Jansen’s medium. Growth was examined after incubation for 5 days in comparison with negative control.

### Antibiotic sensitivity test

Sensitivity test was performed using antibiotic impregnated discs (6 mm diameter). Antibiotic sensitivity of the strains were tested against chloramphenicol, erythromycin, rifampicin, polymixin B by Kirby Bauer disc-diffusion assay method (Kumar et al. 2015). The quantities of antibiotics used were 30 µg/disc. On the basis of inhibition zone recorded, organisms were categorized as resistant or sensitive according to DIFCO Manual 10th edition (1984).

### Antibacterial activity

All endophytes strain were screened for antibacterial properties against *Escherichia coli*, *Pseudomonas aeruginosa* and *Klebsiella pneumonia*. The nutrient agar plates were inoculated with bacterial endophytes as a single streak at the centre of the petri-plate and incubated for 7 days at 30 °C. Overnight grown cultures of the test organisms were streaked at right angle to the producer endophyte and observed for its growth/inhibition after 24–48 h of incubation at 30 °C. The length of inhibition zone was measured to nearest mm (Kumar et al. 2015).

### Antifungal activity

All the endophytic strains were tested against four moulds *Fusarium solani*, *Alterneria alternata*, *Byssochlamys fulva* and *Aureobasidium pullulans* for the fungistatic activity. The 24-h-old cultures of separate strains grown in nutrient broth were spotted on the fungal test cultures prepared on the PDA medium (Kumar et al. 2015). The plates were incubated at room temperature for 7 days and fungal growth inhibition was measured (Owen and Hundley 2004; Kumar et al. 2015).

### Salt tolerance

To assay the salt tolerance of endophytic bacterial isolates a 20 µl aliquots of an 24 h old test culture was inoculated with 1 % protease peptone in a sequential series of 1, 2, 3, 4, 5, 6, 7, 8, 9 and 10 % NaCl concentrations and left incubated under growth condition. After 24–48 h their growth was measured by absorbance at 600 nm in a spectrophotometer (UV/VIS Spectrophotometer117, Systronics, India).

## Results

### Physio-biochemical characterization

A total of 14 different bacterial clones on the basis of colony morphology and colors were isolated from the sliced turmeric rhizome while no bacteria could be observed near the surface sterilized unsliced rhizome. Based on the morphology and biochemical characteristics, isolates were assigned to three genera *Bacillus* (9), *Pseudomonas* (3) and *Clavibacter* (2). Nearly two-third of the bacterial isolates were rod shaped and Gram positive; details of the biochemical characteristics are presented in Table 1.

**Table 1:** Bochemical characterization of selected endophytic bacterial strain of turmeric rhizome

	*Bacillus cereus* ECL1	*Bacillus thuringiensis* ECL2	*Bacillus* sps. ECL3	*Bacillus pumilis* ECL4	*Pseudomonas putida* ECL5	*Clavibacter Michiganensis* ECL6
Gram staining	+ve	−ve	+ve	+ve	−ve	+ve
Shape	Rod	Rod	Rod	Rod	Rod	Rod
Catalase	+ve	−ve	+ve	+ve	+ve	+ve
Oxidase	−ve	−ve	+ve	−ve	+ve	+ve
Glucose	+ve	+ve	+ve	+ve	+ve	+ve
Lactose	−ve	−ve	+ve	+ve	−ve	−ve
Maltose	+ve	−ve	+ve	−ve	+ve	−ve
Mannitol	+ve	−ve	+ve	−ve	−ve	−ve
d-Mannose	+ve	−ve	+ve	−ve	−ve	−ve
Sucrose	+ve	−ve	+ve	+ve	−ve	+ve
Nitrate reduction	+ve	−ve	+ve	−ve	−ve	+ve
H_2_S production	−ve	+ve	−ve	−ve	+ve	−ve
Starch hydrolysis	+ve	−ve	+ve	+ve	+ve	+ve
Salt tolerence	7 %	8 %	7 %	8 %	6 %	6 %

+ve, positive; −ve, negative

**Fig. 1:**
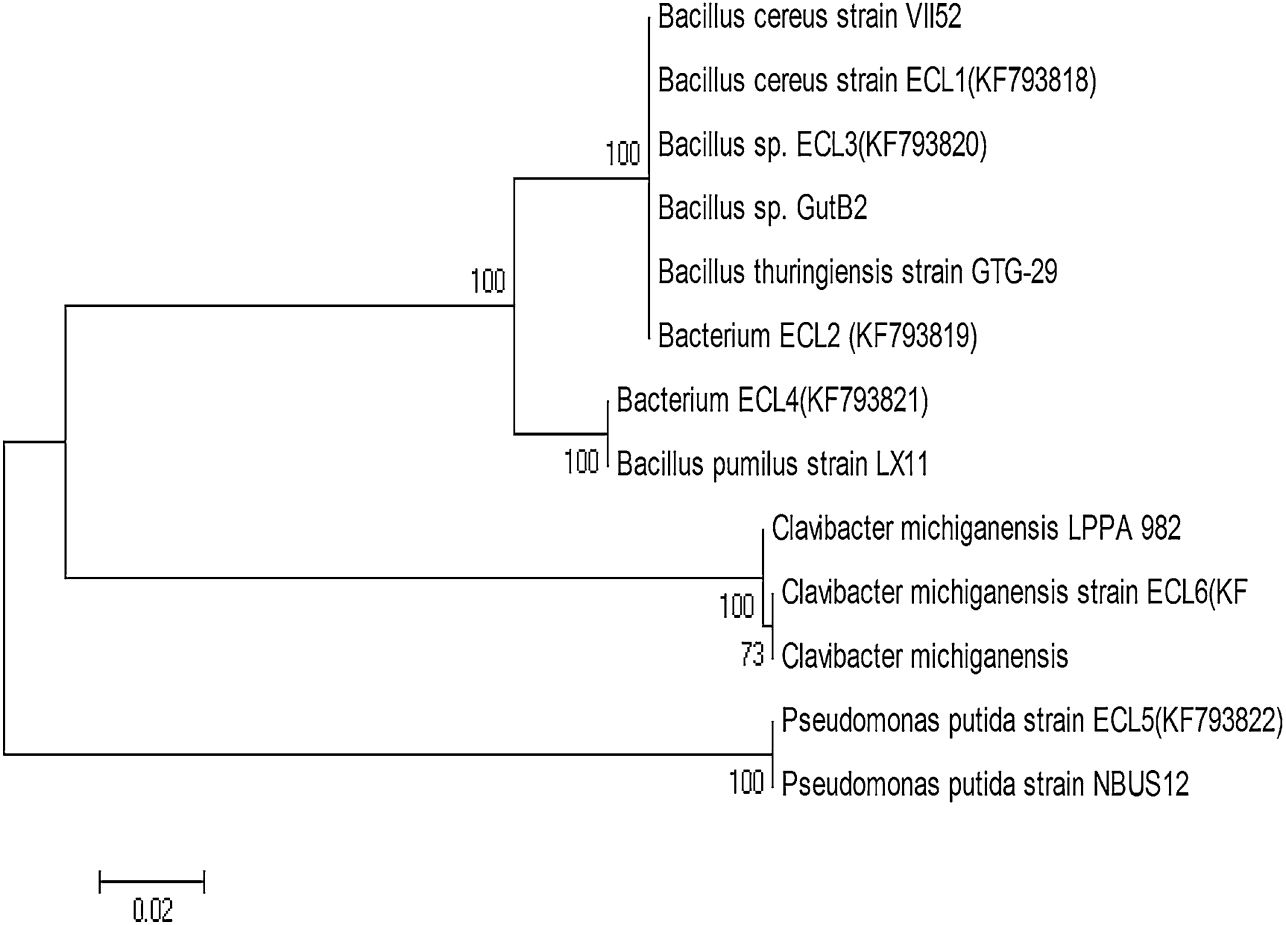
Phylogenetic tree from analysis of 16S rRNA gene sequence of the endophytic strains of *C. longa* strains using neighbor joining approach. Each *number* on a branch indicates the bootstrap confidence values correspond to the *scale bar* of branch lengths. GenBank accession numbers of nucleotide sequences are shown along with the name of bacterial strain. Phylogenetic analyses were conducted in MEGA 5

On the basis of 16S rRNA gene sequence, isolates were identified as *Bacillus cereus* ECL1 (KF793818), *Bacillus thuringiensis* ECL2 (KF793819), *Bacillus* sp. ECL3 (KF793820), *Bacillus pumilus* ECL4 (KF793821), *Pseudomonas putida* ECL5 (KF793822), *Clavibacter michiganensis* ECL6 ((KF793823). Endophytic bacterial strains belonged to Bacteroidates (*Clavibacter)*, Firmicutes *(Bacillus)*, γ-Protobacteria (*Pseudomonas).* The details of the strains and the nearest relative based on 16S rRNA gene sequence are given in Table 2. The DNA sequences were aligned and phylogenetic tree constructed by neighbor-joining method using MEGA5.01 (Fig. 1).

### PGP traits

All the endophytic bacterial strains produced IAA with maximum (23 µg ml^−1^) in *P. putida* (ECL5) and minimum (14 µg ml^−1)^ in *Clavibacter michiganensis* (ECL6) on supplementation of 400 l-tryptophan (µg ml^−1^) and remaining four strains value of IAA were in between them. Siderophore production was observed only in *Bacillus * sp. (ECL3) and *P. putida* (ECL5). All the strains solubilized tricalcium phosphate except *B. thuringiensis* ECL2 (Table 3).

**Table 2:** Closest relative of the isolated strains as revealed by 16S rRNA gene sequencing

S. No.	Source	Strains	Accession number	Nearest phylogenetic neighbour	Similarity (%)
1	*Curcuma longa*	ECL1	KF793818	*Bacillus cereus* VII52	100
2	*Curcuma longa*	ECL2	KF793819	*Bacillus thurigiensis GTG*-*29*	100
3	*Curcuma longa*	ECL3	KF793820	*Bacillus * sp. GutB2	100
4	*Curcuma longa*	ECL4	KF793821	*Bacillus pumilus strain LXII*	99
5	*Curcuma longa*	ECL5	KF793822	*Pseudomonas putida* NBUS12	100
6	*Curcuma longa*	ECL6	KF793823	*Clavibacter michiganensis* LPPA982	100

The endophytes strains showed different level of tolerance to the increasing salt concentration strain *B. thuringiensis* (ECL2) and *B. pumilus* (ECL4) withstand higher salt level (8 % NaCl) where as *B. cereus* ECL1 and *Bacillus* sp. ECL3 tolerated 7 % of NaCl. *Pseudomonas putida* (ECL5) and *Clavibacter michiganensis* (ECL6) bacterial population survived at 6 % of NaCl concentration.

### Carbon and nitrogen source utilization pattern

All the endophytic strains variably utilized glucose, sucrose and yeast extract as a C source. Mannitol was utilized by the strains *B*. *cereus* (ECL1) and *Bacillus* sp. (ECL3), where as no strain utilized mallic acid and methanol as carbon source. All the strains utilized glycine, alanine, cystine and glutamine as N-source, *P. putida* ECL5 also utilized aspartic acid and glutamic acid. Ammonium sulphate was utilized by all the isolates except *C*. *michiganensis* (ECL6), Arginine is utilized by four strains except *B*. *cereus* (ECL1) and *Bacillus* sp. (ECL3).

**Table 3:** Characterization of selected bacterial isolates for plant growth promoting (PGP) traits

	IAA production	P. solubilization	Siderophore production
*Bacillus cereus* ECL1	+ve	+ve	−ve
*Bacillus thuringiensis* ECL2	+ve	−ve	−ve
*Bacillus * sp. ECL3	+ve	+ve	+ve
*Bacillus pumilis* ECL4	+ve	+ve	−ve
*Pseudomonas putida* ECL5	+ve	+ve	+ve
*Clavibacter michiganensis* ECL6	+ve	−ve	−ve

### Antibiotic sensitivity

Antibiotic sensitivity pattern of the endophytic bacterial isolate was determined against four different antibiotics by disc diffusion method. Results shown in Table 4 depict that endophytes of turmeric were mostly sensitive to chloramphenicol followed by erythromycin while resistant to rifampicin and polymixin-B. *Bacillus thuringiensis* (ECL2) and *B. pumilus* (ECL4) showed high resistance for two (rifampicin and polymixin-B) of the four used antibiotics whereas strains *B. cereus* (ECL1) and *Bacillus * sp. (ECL3) were most susceptible to all the antibiotic disc.

**Table 4:** Antibiotic sensitivity, antibacterial activity and antifungal activity of endophytic strains of *C. longa* L.

Bacterial strain	Antibiotic sensitivity (antibiotics inhibition zone in mm)				Antibacterial activity (inhibition zone in mm)			Antifungal activity (fungal growth inhibition)			
	Chloramphenicol	Erythromycin	Rifampicin	Polymixin-B	*Escherichia coli*	*P. aeruginosa*	*K. pneumoniae*	*F. solani*	*A. alternata*	*B. fulva*	*A. pullulans*
ECL1	26(S)	22(S)	16(S)	13(S)	+13	−	+11	**+**	**+**	**+**	**+**
ECL2	23(S)	19(S)	14(S)	0(R)	+14	−	−	**+**	**+**	**+**	**+**
ECL3	25(S)	20(S)	13(S)	11(I)	+11	−	+10	**+**	**+**	−	−
ECL4	19(S)	17(S)	0(R)	0(R)	+10	−	+9	+	+	+	+
ECL5	22(S)	19(S)	11(1)	12(I)	+16	−	+14	+	+	+	+
ECL6	21(S)	16(S)	12(I)	0(R)	+9	−	−	+	+	−	−

+, presence of activity

−, absence of inhibition zone

### Antibacterial activity

Antibacterial properties of all bacterial endophytes were assessed against three bacterial test organisms, *E. coli*, *P. aeruginosa* and *Klebsiella pneumoniae*. The isolate which inhibited growth of any of the test organism(s) was considered having antibacterial activity and the length of inhibition zone was measured in mm (Table 4). All the strain showed antibacterial activity against *E. coli*, where as strain *B. cereus* ECL1, *Bacillus* sp. (ECL3), *B. pumilus* (ECL4), *C. michiganensis* (ECL6) showed antibacterial activity against *K. pneumonia* whereas no any strains inhibits the growth of *P. aeruginosa*. Strain *B. cereus* (ECL1) and *P. putida* (ECL5) possessed strong antibacterial property among all the tested strain.

### Antifungal activity

To assess fungistatic property of the bacterial strains, four fungal species namely *F. solani*, *Alternaria alternata*, *B. fulva* and *A. pullulans* were used. Fungistatic activity was observed by zone of growth inhibition in the area where the bacteria were inoculated on the agar plate. All the strains exhibited antifungal property but *Bacillus* sp. (ECL3) did not show fungistatic activity against *B. fulva* and *A. pullulans* strain (Table 4).

## Discussion

The turmeric rhizome was rich in endophytic bacterial diversity. In the turmeric, isolated endophytic bacterial isolates belonged to six different species *B. cereus* (ECL1), *B. thuriengenesis* (ECL2), *Bacillus* sp. ECL3, *B. pumilus* (ECL4), *P. putida* (ECL5) and *C. michiganensis* (ECL6). These strains previously reported as endophytes in different plant species like *Bacillus* sp. and *Pseudomonas* sp. in tomato and rhizomes of ginger (Rashid et al. 2012; Jasim et al. 2013). *B. cereus* isolated from C*henopodium majus* (Goryluk et al. 2009), P*. putida* in *Zingiber officinale* (Jasim et al. 2013) and *Poulus* tree (Taghavi et al. 2009). *C. michiganensis* strain reported as a pathogen which cause cankar of tomato and goss wilt of corns but it is also reported as non-pathogenic in the Prairie plants (Zinniel et al. 2002). Pathogenesis is a multi-factorial process which depends on the immune status of the host, ability to invade host tissue and plants exudates. Turmeric endophytic isolates *C. michiganensis* (ECL6) was non-pathogenic in nature, this may be due to the properties of biochemical constituents curcuminoids and sesquiterpenoids present in the plant which have antimicrobial, antifungal, antioxidant properties. In the previous study of Ding et al. (2011) also used *Calvibacter* sp. as inoculants in *Chorispora bungeana* for chilling tolerance. During the plant growth promotion trait analysis, all the endophytic strains produced significant amount of IAA, which has already been reported in *B. cereus* (Rana et al. 2011) and *P. putida* (Jasim et al. 2013). The extent of production was found maximum in case of *P. putida* (ECL5) and minimum in *C. michiganensis* (ECL6) in the presence of tryptophan. IAA is the most common plant hormone, which stimulate the growth and reproduction of plants (Taghavi et al. 2009). IAA produced by bacteria interacts with the plants in diverse ways from pathogenesis to phytostimulation. IAA is the main auxin in the plants which involved in cell enlargement and division, tissue differentiation, physiological processes (Woodward and Bartel 2005; Spaepen et al. 2007). The amount of IAA produced by bacteria play important role in plant–microbe interaction (Xie et al. 1996). The modulation of plant growths takes place by optimal IAA concentration range. In the study of Persello-Cartieaux et al. (2003) it is found that inoculation of IAA producing bacteria *Pseudomonas thivervalensis* at the amount 10^5^ CFU ml^−1^ in *Arabidopsis* resulting reproducible morphological changes but the amount of 10^6^ CFU ml^−1^ inoculants inhibit the plant growth.

Siderophore production by the bacterial strain is one of the biocontrol mechanism. The iron-chelation by bacteria makes them better competitors for the available iron and in this way, prevents growth of the pathogenic microorganisms. In this study siderophore production was observed only in two strains *Bacillus* sp. (ECL3) and *P. putida* (ECL5), similar to the previous report by Jasim et al. (2013). Plant growth promoting bacteria solubilize insoluble phosphates to make them available to enhance crop productivity. Four endophytic strains *B. cereus* (ECL1), *Bacillus* sp. (ECL3), *B. pumilus* (ECL4) and *P. putida* (ECL5) solubilized phosphate which strengthen the results as reported previously in *Bacillus* sp. and *P. putida* (Pandey et al. 2006; Forchetti et al. 2007).

The endophytic bacterial isolates reside and multiply in the plants where the environment contains relatively high ionic strength which successively tolerated both the biotic and abiotic factors. Previously many authors reported the endophytic strain which successively tolerated the high salt concentration (Hallmann et al. 1997a, b; Lopez et al. 2011; Rashid et al. 2012; Singh et al. 2013; Kumar et al. 2015). In this study the endophytic isolates were able to grown differentially at different salt levels. In a previous study, *Pseudomonas* sp. tolerated up to 4 % NaCl, while *Bacillus* sp. 2 % NaCl (Rashid et al. 2012). The endophytic bacterial strains of *Momordica charentia* showed tolerance to 4–10 % NaCl (Singh et al. 2013).

Many endophytic bacterial strains exhibited antibiotic properties that inhibit the growth of an antagonistic bacterium. Amongst all bacterial strains were sensitive to Chloramphenicol and Erythromycin but the strain *B. thuriengenesis* (ECL2), *B. pumilus* (ECL4) resistant to polymixine-B and rifampicin respectively. The antibiotic disc acts differentially on the growth of same bacterial strains of different isolation source. In case of *Cassia tora* bacterial isolate *Pseudomonas* sp. resistant to chloramphenicol and amoxicillin (Kumar et al. 2015) in contrast to *Pseudomonas* species of *Andrographis paniculata* which showed resistance only to amoxycillin (Arunachalam and Gayathri 2010). Many natural products produced by endophytes have proven to be antibacterial, antifungal, antidiabetic, antioxidants and immunosuppressives and great source of bioactive natural products. The majority of endophytic bacteria produced different kinds of novel antibiotics like Ecomycins, Pseudomycins, Munumbicins, Kakadumycins. These compounds inhibit the growth of pathogenic bacteria and fungi (Christina et al. 2013).

The bacterial strains secrete different types of natural products to inhibit or kill a wide variety of harmful disease-causing agents including, bacteria, fungi, viruses and protozoans that affect humans and animals (Demain 1981). The bacterial secretes 2,4-diacetylphloroglucinol (DAPG), phycocyanin, siderophores, lytic enzymes, chitenase which degrade the cell wall of pathogens and act as natural biological control. In present investigation the strain *B. cereus* (ECL1) and *P. putida* (ECL5) have shown the inhibition zones and indicated a strong antibacterial property among the all isolates.

During the antifungal activity the tested fungal strain are potent pathogens and cause severe infection to living organisms. *Fusarium solani* is a filamentous fungus commonly isolated from the soil and plant debris. *A. alternata* is known to produce mycotoxins, *A. pullulans* and *B. fulva* are responsible for fruit rot in certain plants. The antifungal activity of the isolated strains is due to the secretes of the strains like lytic enzymes, chitenase, production of certain antibiotics. Our finding showed that all bacterial isolates posses antifungal characteristics against above pathogens except the strain *Bacillus* sp. (ECL3) and *C. michiganensis* (ECL6), which did not show fungistatic activity against the fungal strain *B. fulva* and *A. pullulans* and they may be helpful to host for resistance against virulent fungi.

## Conclusion

The diverse endophytic bacterial strains (ECL1, ECL2, ECL3, ECL4, ECL5, ECL6) are isolated from the rhizome of *C. longa*. They harbor PGP traits of variable degree to accomplish the need of the host. All the strains produced IAA; two-third solubilized phosphate while one-third produced siderophore and tolerated high salt (8 % NaCl) concentration during salinity tolerance. All the bacterial strains differentially utilized the carbon and nitrogen source. The strains were sensitive to antibiotic chloromphenicol followed by erythromycin where as most of them were resistant to polymixine B. The endophytic strains effectively checked the growth of *E. coli*, *Enterobacter* and some of the fungal strain like *Fusarium solani* and *A. alternata*.

## Electronic supplementary material

Below is the link to the electronic supplementary material.

Supplementary material 1 (JPEG 761 kb)
