# Supplementary figures and images for: Isolation and characterization of bacterial endophytes of Curcuma longa L
Source: 3 Biotech. 2016 Feb 13;6(1):60. doi: 10.1007/s13205-016-0393-y (PMC4752947; doi:10.1007/s13205-016-0393-y)

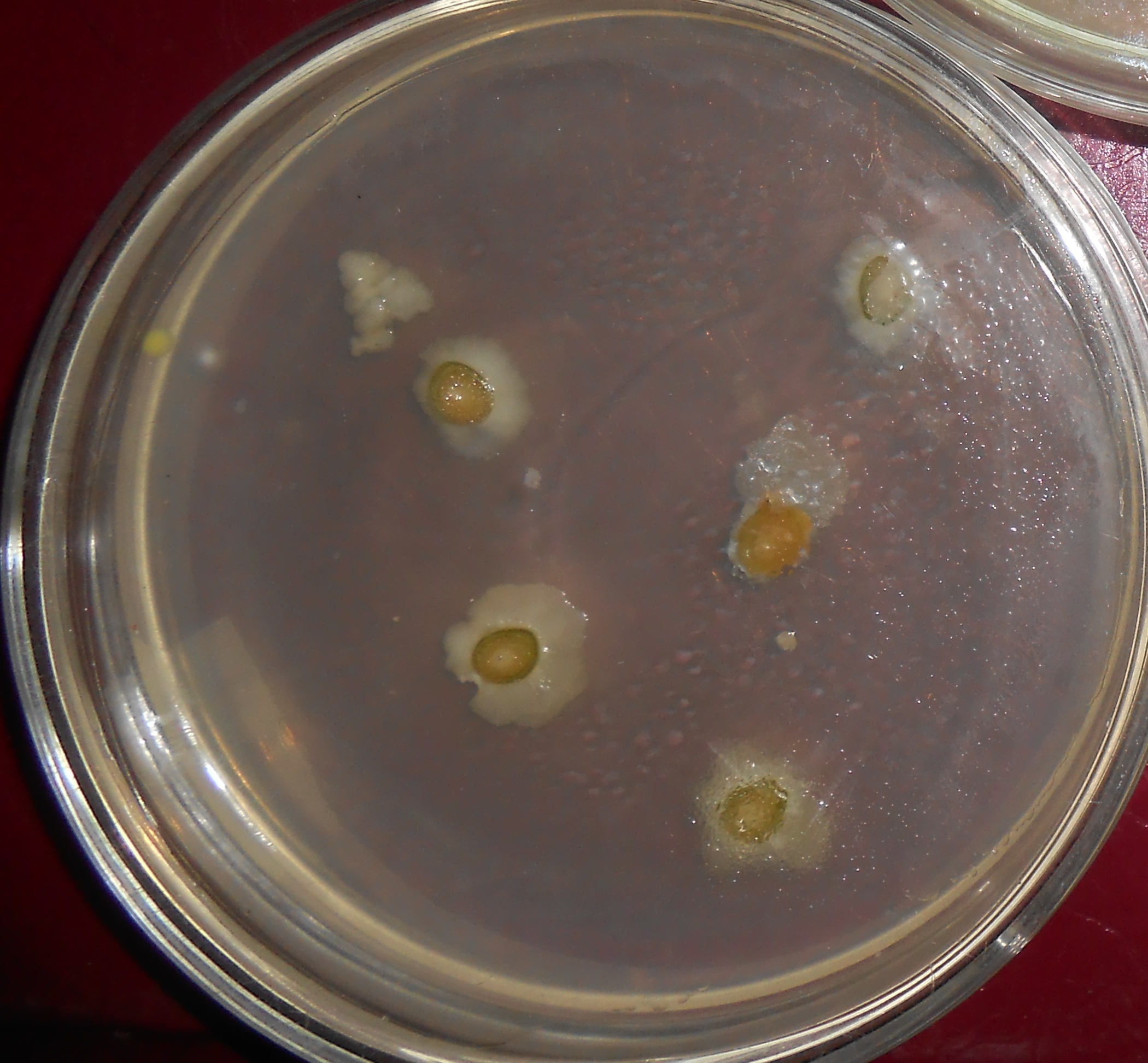

Supplement: Supplementary file 1 — Supplementary material 1 (JPEG 761 kb) [file 13205_2016_393_MOESM1_ESM.jpg]
